# Superatomic states under high pressure

**DOI:** 10.1016/j.isci.2023.106281

**Published:** 2023-03-01

**Authors:** Rui Wang, Xinrui Yang, Wanrong Huang, Zhonghua Liu, Yu Zhu, Hanyu Liu, Zhigang Wang

**Affiliations:** 1 Institute of Atomic and Molecular Physics, Jilin University, Changchun 130012, China; 2 International Center for Computational Method & Software and State Key Laboratory of Superhard Materials, College of Physics, Jilin University, Changchun 130012, China; 3 Institute of Theoretical Chemistry, College of Chemistry, Jilin University, Changchun 130023, China

**Keywords:** Atomic physics, Atomic properties, State of matter

## Abstract

The study of superatoms has attracted great interest since they apparently go beyond the traditional understanding of the periodic table of elements. In this work, we clearly show that superatoms can be extended from conventional structures to states under pressure condition. By studying the compression process of the CH_4_@C_60_ system formed via embedding methane molecules inside fullerene C_60_, it is found that the system maintains superatomic properties in both static states, and even dynamic rotation situations influenced by quantum tunneling. Remarkably, the simulations reveal the emergence of new superatomic molecular orbitals by decreasing the confined space to approach the van der Waals boundary between CH_4_ and C_60_. Our current results not only establish a complete picture of superatoms from ambient condition to high pressure, but also offer a perspective for the discovery and exploration of new properties in superatom systems under extreme conditions.

## Highlights

• Superatoms can be extended from conventional structures to states under pressure
• Under isotropic compression, the CH_4_@C_60_ maintains superatomic properties
• The new SAMOs appear as the space between CH_4_ and C_60_ is reduced to the vdW boundary

## Graphical abstract

**Figure:**
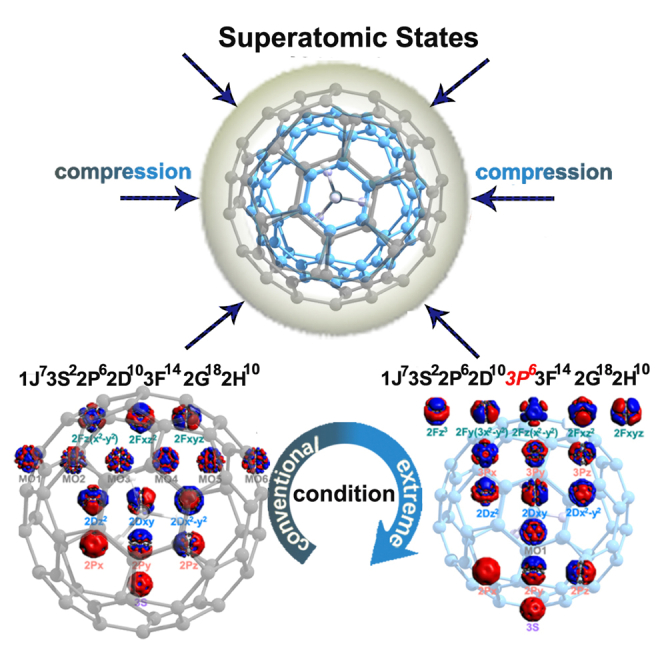

## Introduction

Superatom has attracted significant attention, since it exhibits novel physical and chemical properties, which should be of fundamental interest in the fields of physics, chemistry, and material science. The characteristic of superatoms is often determined by the atom-like electronic arrangement.^1^^,^^2^^,^^3^^,^^4^^,^^5^^,^^6^^,^^7^^,^^8^^,^^9^^,^^10^ Mass spectrometry experiment is a key step to identify superatom^3^ and the magic number rule for electrons is gradually summed according to the jellium model.^1^^,^^3^^,^^4^^,^^5^ However, there is a concern about the stability of superatoms under convention conditions in the past, and even more on their isolated structures.^11^^,^^12^^,^^13^^,^^14^^,^^15^^,^^16^^,^^17^^,^^18^^,^^19^

At present, it is well known that extreme conditions, especially high pressure, are the common means to create exotic properties that are not accessible at conventional conditions, by influencing the microscopic structure, the interatomic interactions and so on.^20^^,^^21^^,^^22^^,^^23^^,^^24^^,^^25^ For example, the predicted near-room-temperature superconductivity in LaH_10_ is experimentally confirmed under high pressure.^26^^,^^27^^,^^28^^,^^29^ The emergence of the remarkable properties under high pressure is often found to violate the traditional understanding from structures to states.^21^^,^^22^ We propose a supposition as to whether the superatoms, as a special class of molecules, can also achieve the transformation from the structure to the state under extreme conditions. This will open up the exploration of superatom in non-ambient environment to enrich the development of superatomic concept and extend their dimension, which could greatly bring new prospects for the efforts to expand the periodic table of superatomic elements and bottom-up construct materials or devices.^7^^,^^30^^,^^31^^,^^32^ We also hope that the reason for the existence of special properties under high-pressure can be explained at the atomic level from the superatomic perspective to promote the development of interdisciplinary studies.

Regarding this issue, it is necessary to study such systems which are of interest in both superatoms and high-pressure field. Fortunately, there are few systems as suitable as fullerene C_60_ and its derivatives.^16^^,^^33^^,^^34^^,^^35^^,^^36^^,^^37^^,^^38^ On the one hand, the molecular configuration of single C_60_ has be obtained in low-temperature simple cubic phase under hydrostatic pressure by the pressure-transmitting-fluid, in which its covalent interaction of C-C bond is enhanced and lattice constant hardly change compared to that of face-centered-cubic phase under general conditions. This realizes isotropic compression.^39^^,^^40^^,^^41^^,^^42^ On the other hand, the derivatives of C_60_ can reveal interesting physical and chemical properties after encapsulating atoms or molecules based on its unique confined environment, so that they have potential applications in molecular switches and expected metallic or even superconducting character.^38^^,^^43^^,^^44^^,^^45^^,^^46^^,^^47^ The interactions between the confined environments and the embedded group have various ranges from general intermolecular interactions to interactions within van der Waals boundaries, offering the new physical phenomena, such as anomalous enhancement of molecular rotation, pressure-induced change of metallic properties.^48^^,^^49^^,^^50^ Therefore, endohedral fullerenes may offer a suitable platform for the study of static superatom properties, especially, the enhanced connection between the inner guest and the outer cage during the compression process, and their dynamic mean state under high pressure. Since the movement of light nuclear elements contained by methane in a confined environment is also attracting much attention, the CH_4_@C_60_, as a typical experimentally confirmed system,^46^^,^^51^ providing a unique opportunity to explore the superatom properties of the system under compression.

In this work, we take the CH_4_@C_60_ system as a representative example to investigate the role of pressure in dealing with superatoms. Based on first-principles calculations, we found the superatomic properties in the compression process from general intermolecular interactions to interactions within the van der Waals boundaries between CH_4_ and C_60_. There are changes in the arrangement of superatomic molecular orbitals (SAMOs) and degree of MOs’ delocalization. It includes that the localized MOs contributed by CH_4_ are gradually converted into SAMOs, accompanied by the generation of 3P-SAMOs. Obviously, these phenomena cannot stand when the compressive condition is removed. This represents a novel superatom physical state under pressure instead of the traditional picture. Our work provides new insights for future research toward the discovery of the physical properties and application of superatoms.

## Results and discussion

### The compression process of CH_4_@C_60_

**Figure 1:**
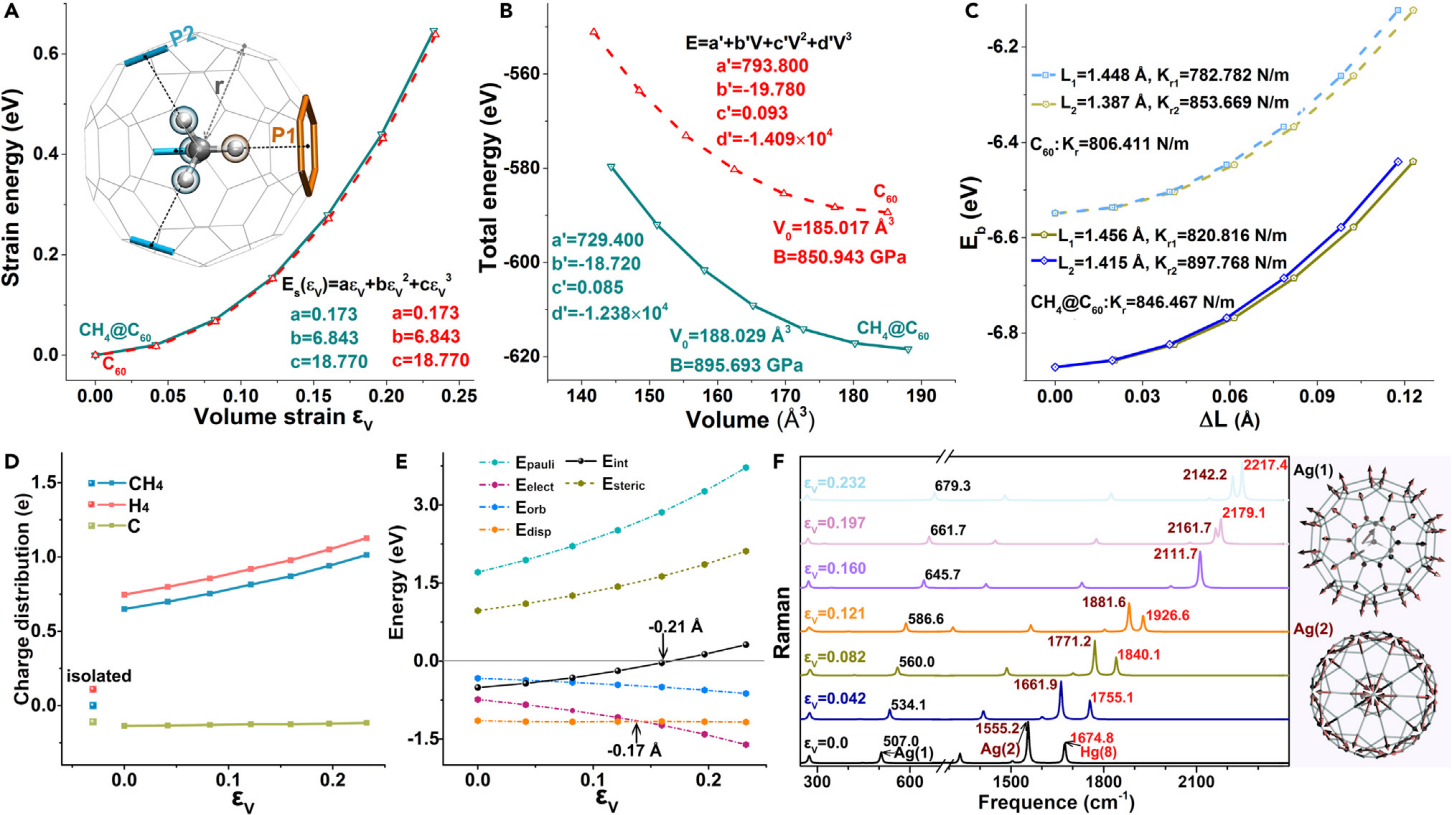
The compression process of CH_4_@C_60_ (A) The strain energy (E_S_) as a function of the (ε_V_) for the CH_4_@C_60_ and C_60_. The schematic diagram of the stable structure based on the established coordinate system is shown. Embedded methane has two types of H atoms pointing to different positions. One points to the center of aromatic ring (P1) on C_60_. The other points to the common edge (P2) of two aromatic rings on C_60_. (B) The total molecular energy (E) as a function of the volume (V) for the CH_4_@C_60_ and C_60_. In (A) and (B), the polynomial fitted according to the curves is presented. (C) The binding energy (E_b_) as a function of variation of bond length (L_b_) for the CH_4_@C_60_ and C_60_. Due to the C_60_ have two kinds of C-C covalent bonds, the b values contain 1 and 2. (D) The charge distribution of embedded methane during compression. Here, the charge distribution of isolated methane is also given. (E) The EDA of interaction between CH_4_ and C_60_ during compression. (F) Raman spectrum of CH_4_@C_60_ during compression. The active vibrational modes, Ag(1) and Ag(2), are marked.

We began our simulation on the structural optimization of the CH_4_@C_60_, which shows the C_3v_ symmetry (Figure 1A, Table S1). Given that these structures have one *C3* symmetric axis, four H atoms have two typical positions as shown in Figure 1A. The average distance between the C atom on the methane molecule and the C atoms on the C_60_ cage of CH_4_@C_60_ is called the radius of fullerene (r). The radius of initial fullerene (r_0_) is 3.554 Å, which is coincided with the previous experimental result.^46^ To simulate isotropic hydrostatic compression resulting from pressure-transmitting-fluid of experiment, the equal proportional compression of fullerenes was explored. The volume strain ($\epsilon_{V}$) is defined as 1-r^3^/r_0_^3^, which varies from 0.0 to 0.232 upon compression. To study the physical properties being of concern on fullerene under high pressure, the effective strain energy constant (E″), bulk modulus (B), and bond force constant (k_r_) for the CH_4_@C_60_ were calculated (the details show in Figures 1A–1C). The results show that compared with C_60_ (E″ = 22.643 eV/atom, B = 850.943 GPa, k_r_ = 806.411 N/m), CH_4_@C_60_ have higher E″ (22.928 eV/atom), B (895.693 GPa) and k_r_ (846.467 N/m). It is consistent with the results summarized that the B and E″ values of the endohedral fullerene than that of the fullerene and may correlate with higher density corresponding to higher mechanical properties.^52^^,^^53^^,^^54^^,^^55^ While the higher k_r_ for endohedral fullerene could be explained by comparing the bond lengths between the carbon atoms.^52^ These calculations imply that the embedded methane also tends to increase the pressure resistance and rigidity of the fullerene.^52^

As a basis of superatomic state exploration, it is fundamental to discuss the change in the electronic structure of superatoms under pressure. The charge distributions of cage and the embedded molecule by Voronoi deformation density (VDD)^56^ method were analyzed. As shown in Figure 1D and Table S2, it can be clearly seen that the amount of negative charge transferred from CH_4_ to C_60_ increases as the compression continue, which comes mainly from the H atoms. In addition, the dramatic increase in the tendency of H atoms to lose negative charges in CH_4_@C_60_ compared to isolated methane and the almost no charge transfer between C atoms for isolated C_60_ both suggest that intercalated methane induces charge transfer. To ensure the reliability of the above results, Hirshfeld^57^ method was also employed, and the results are qualitatively consistent with VDD (Table S3). To provide detailed insights into the intermolecular interaction between CH_4_ and C_60_ during compression, energy decomposition analysis (EDA) was carried out. Total interaction (E_int_), Pauli repulsion interaction (E_Pauli_), three attraction interactions, containing the electrostatic (E_elect_), orbital (E_orb_), and dispersion (E_disp_) interactions as a function of $\epsilon_{V}$ are shown in Figure 1E. For the attraction interaction of the equilibrium structures, the E_disp_ is dominant, illustrating that interaction between two components is typical van der Waals. While in the compression process, the E_elect_ becomes more important and exceeds the E_disp_ corresponding with the Δr of about −0.17 Å. That is, from this point onwards, the classical coulomb interaction account for the largest proportion of total attractive interactions, rather than van der Waals interaction.^58^ Furthermore, the E_int_ changes from negative to positive value as the Δr is −0.21 Å, which suggests that interaction between the CH_4_ and C_60_ are within van der Waals boundary.

### The Raman spectrum characterization of CH_4_@C_60_ during compression

Given the importance of vibration-related spectra on experimental observation, the Raman spectrum of CH_4_@C_60_ was calculated. For CH_4_@C_60_, as shown in Figures 1F and S1, there are three kinds of Raman active vibrational modes, containing radial breathing, tangential pentagonal pinch modes on the cage (marked as Ag(1) and Ag(2)), and a tensile vibration of C-C covalent bonds (marked as Hg(8)). Interestingly, the Ag(2) and Hg(8) modes are unable to distinguish when the $\epsilon_{V}$ is 0.160. This phenomenon also can be seen in the Raman spectrum of isolated C_60_ (Figure S2). Actually, it has been observed in the experimental research of C_60_ at a pressure of 26 GPa.^24^ The merging phenomenon between the Ag(2) and Hg(8) modes can be used to determine whether or not CH_4_ and C_60_ are within the van der Waals boundary, thus greatly facilitating the analysis of changes in superatomic state in the future experiments.

### The electronic configuration analysis of CH_4_@C_60_ during compression

**Figure 2:**
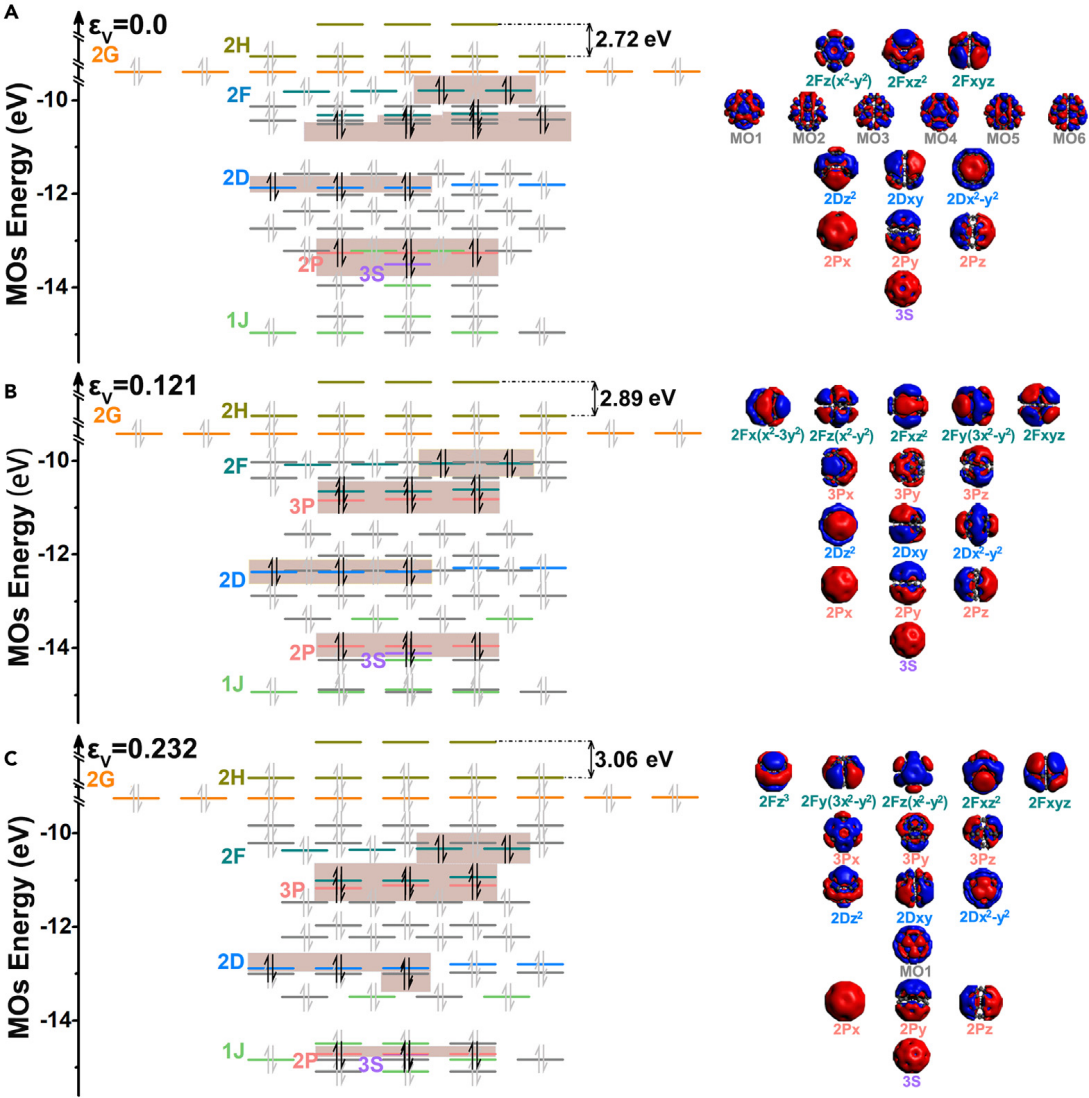
The diagrams of energy levels during compression (A)–(C) display the energy levels diagrams and part molecular orbitals (MOs) when the $\epsilon_{V}$ is 0.0, 0.121, and 0.232, respectively. Here, the MOs energies range from −15.6 to −3 eV. The SAMOs with different angular momenta are marked by different colors and the HOMO-LUMO gaps are presented. The MOs, which are both contributed by the embedded methane and cage, are marked by black arrows and shadow, and the corresponding MOs are shown on the right.

The occupied delocalized MOs were analyzed to confirm whether the electron configuration of the superatom can be maintained under the pressure environment, which is an important link to characterize the characteristics of superatoms. Thus, the energy levels and MOs of structures during compression are analyzed as shown in Figures 2 and S3. For uncompressed structure, the electronic configuration of CH_4_@C_60_ is 1S^2^1P^6^1D^10^1F^14^1G^18^1H^16^2S^2^1H^6^1I^26^1J^5^3S^2^2P^6^1J^2^2D^10^2F^14^2G^18^2H^10^ similar to the isolated C_60_.^43^^,^^59^^,^^60^ There is no change in the valence shell of compressed structures (Figures 2B and 2C), indicating that the chemical properties change little under the environment of compression or embedded molecules. The unoccupied S and P SAMOs orbitals also show this point and their enhanced delocalization during compression (Figure S3 and Table S4). In addition, the highest occupied molecular orbital (HOMO)-lowest unoccupied molecular orbital (LUMO) gap becomes larger during compression. It suggests that the system still has good chemical stability.

Furthermore, when the $\epsilon_{V}$ increases to 0.232, there is a change in the generation of 3P SAMOs. Compared with the uncompressed structure, 3P is the new-emerging SAMOs in compressed structures as shown in Figures 2B and 2C. Actually, it begins to be generated when the $\epsilon_{V}$ increases (Figure S4). From analysis on density of state (Figure S5), the MOs belong to the changed electronic configuration are contributed by both methane and C_60_, which are highlighted in right of Figure 2. More strikingly, for the highlighted MOs, there are some regular MOs (MOs1-6) in non-compressed structure. As the r decreases, MOs1-6 are petered out and replaced by SAMOs gradually. This indicates that MOs become more delocalized upon compression. When the $\epsilon_{V}$ is 0.042, these MOs are replaced by new SAMOs of 3P_x_, 3P_y_ and 3P_z_. Then, the ${2F}_{{y(3x}^{2}{-y}^{2})}$ and ${2F}_{{xz}^{2}}$ SAMOs are added when the $\epsilon_{V}$ is 0.121. These SAMOs, including 3S, 2P_x_, 2P_y_, 2P_z_, ${2D}_{z^{2}}$, 2D_xy_, ${2D}_{{x^{2}-y}^{2}}$, 3P_x_, 3P_y_, 3P_z_, ${2F}_{z^{3}}$, ${2F}_{{y(3x}^{2}{-y}^{2})}$, ${2F}_{{xz}^{2}}$, ${2F}_{z\left({x^{2}-y}^{2}\right)}$ and 2F_xyz_, are existed in subsequent compression of the $\epsilon_{V}$ from 0.160 to 0.232. There is the emergence of new superatomic molecular orbitals when the confined space decreases to approach the van der Waals boundary ((Δr is about −0.20 Å). From the geometric and electronic structure of CH_4_@C_60_, the superatoms have the change in the interaction analysis and charge transfer under external conditions of high pressure, but the superatomic properties, the atom-like characteristics of MOs, are maintained, which are described as the superatomic state. The these SAMOs generation is firstly caused by the enhanced electron correlation between methane and cage from the charge transfer and intermolecular interaction analyses. More importantly, there is a crucial factor that the interaction between the two parts enter the van der Waals boundary upon the compression, resulting in a new orbital combination with the two parts. To clarify the composition of the orbitals, the contribution ratio of the cage and methane to the orbitals highlighted in Figure 2 was collected (Table S5), which can also be adapted to the above result.

For exploring the importance of changes in properties brought about by compression, the ultraviolet-visible (UV-Vis) absorption spectra were performed as shown in Figure S6 and Table S6. When the $\varepsilon_{V}$ is 0.232, new sources of absorption peaks are due to the exchange of energy between the F SAMOs and the regular orbitals, resulting in that the latter can be allowed to excited. Moreover, the spectra exhibit interesting phenomena containing the blue-shifted and hyperchromic effect of peaks, which provides a theoretical basis for experimentally fingerprinting the compression.

### The potential energy surface of methane rotation in cage

**Figure 3:**
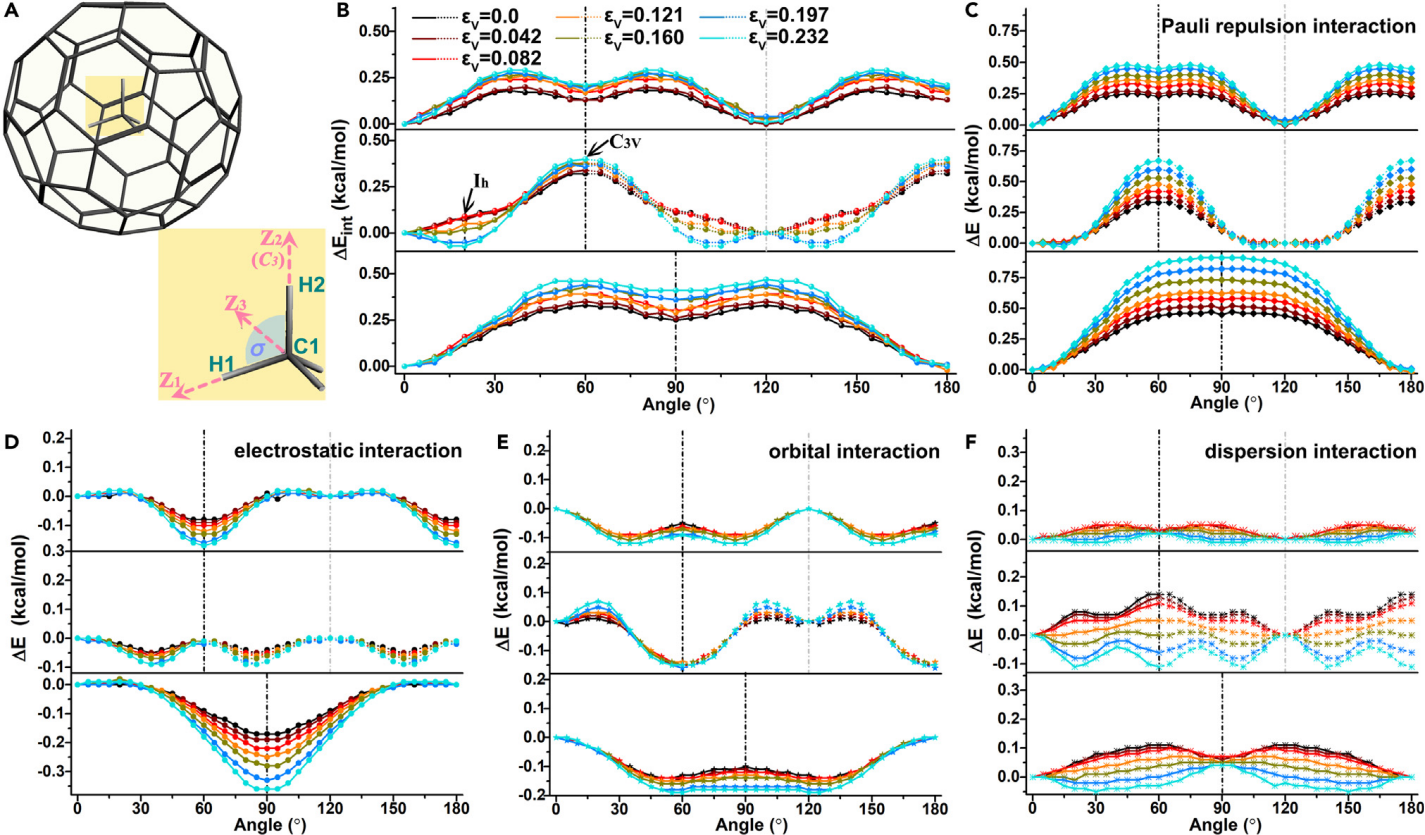
The relative rotation between methane and C_60_ cage during compression (A) The CH_4_@C_60_ diagram. The three rotational axes are the C1-H1 bond (a *C3* symmetric axis of the system), C1-H2 bond, and angular bisectors of ∠H1-C1-H2 marked as Z_1_, Z_2_, and Z_3_. The σ is the plane of reflection symmetry. (B–F) The energy variation of E_int_, E_Pauli_, E_elect_, E_orb_ and E_disp_ between the C_60_ and methane with angle of the methane rotated from 0° to 180°. In each diagram, the top, middle, and bottom are the energy variation rotating around Z_1_, Z_2_, and Z_3_ axes, respectively.

The experimental measurement of molecular states can characterize not only the electron cloud related to the wave function, but also the average dynamic behavior over time. Therefore, we analyzed the rotational behavior of methane and the influence of its interaction with C_60_ on the superatomic state (Figure 3A). The E_int_ curves show the potential energy surface of methane rotation in Figure 3B and they have a certain symmetry and periodicity (Figure S7). From decomposed components curves in Figures 3C–3F, the main source of energy surfaces is the E_Pauli_, while the E_orb_ prevents the formation of energy barrier during rotation. This could explain the reduction of force repulsion caused by the charge transfer during compression. While E_disp_, unlike the other components, varies nonmonotonic with increasing of compression as shown in Figure 3F. When the distance between C_60_ and CH_4_ is compressed to the van der Waals boundary (r of 3.354 Å), the fluctuation of the E_disp_ curves is minimal. At the current stage, structures with I_h_ (rotating to 20° around Z_2_ axis) and C_3v_ (un-rotating structures) symmetry have almost no energy difference (middle of Figure 3B). Further compression leads to the appearance of potential well, which is mainly contributed by the completely opposite change trend of E_disp_ in the early and late stages of compression due to the van der Waals boundary. Another reason why the potential well appears only in Z_2_ is that the isotropy of the rotation of the three H atoms leads to a superposition effect. It illustrates that the methane will spontaneously rotate under pressure.

### The electronic structure analysis of the system during methane rotation

**Figure 4:**
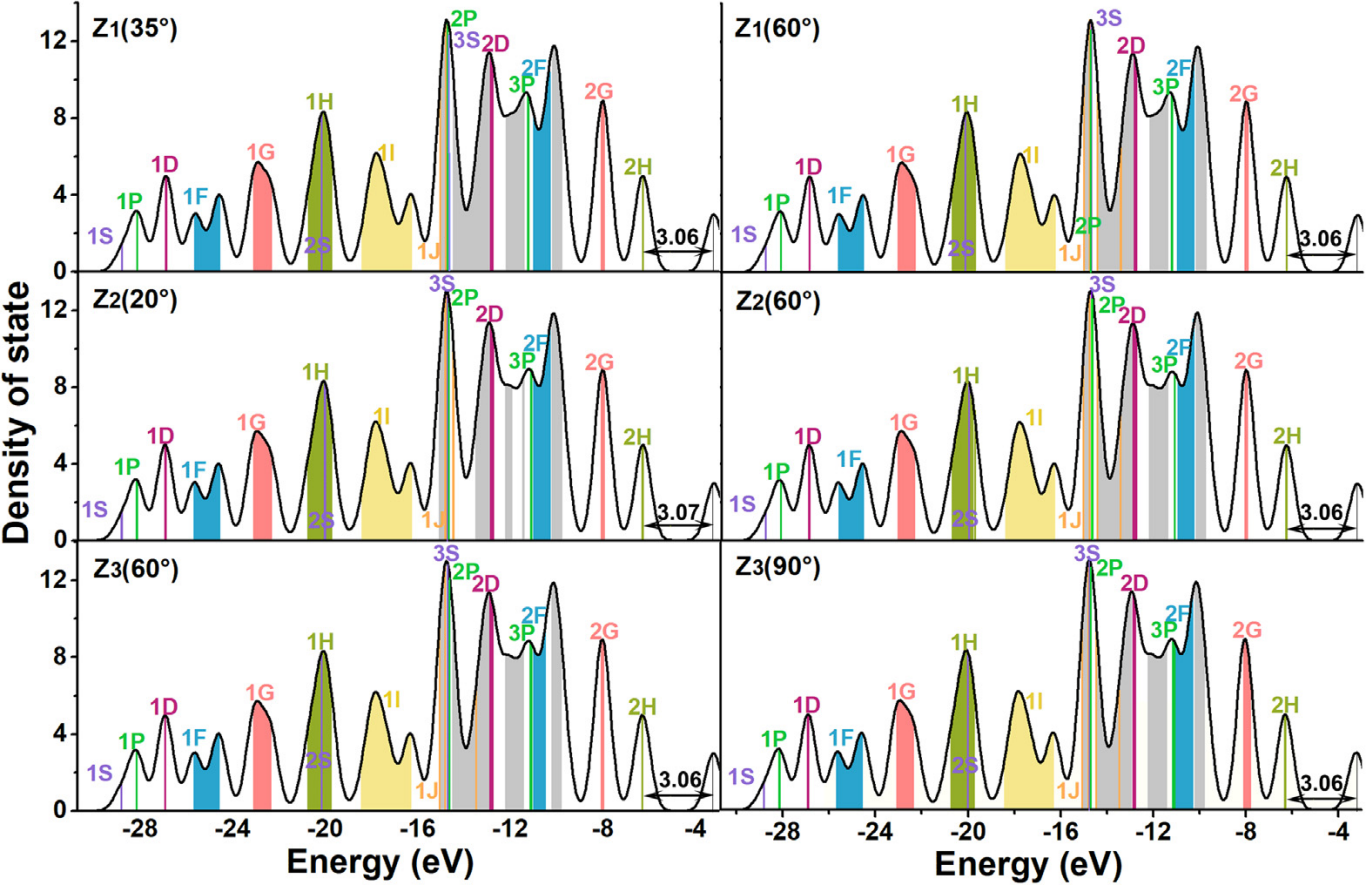
The analyses of DOS during the rotational process These diagrams correspond the rotational angle of 35° and 60° around Z_1_, 20° and 60° around Z_2_, and 60° and 90° around Z_3_, respectively. SAMOs with different angular momenta are marked by different colors. Gray color represents the general molecular orbitals. Here, the $\epsilon_{V}$ is 0.232. The HOMO-LUMO gaps are also indicated (in unit eV).

For further studying the effects of rotational behaviors on superatomic properties, we selected several structures in the rotation process of the maximum compression and analyzed their electronic structures containing the DOS and SAMOs (Figure 4). Comparing with the initial structure, their electronic configuration, 1S^2^1P^6^1D^10^1F^14^1G^18^1H^22^2S^2^1I^26^1J^7^3S^2^2P^6^2D^10^3P^6^3F^14^2G^18^2H^10^, and the HOMO-LUMO gaps (range of 3.06-3.07 eV) almost have no change. These results reveal the existence of the superatomic state in the rotational process.

### The quantum tunneling effect during methane rotation

**Figure 5:**
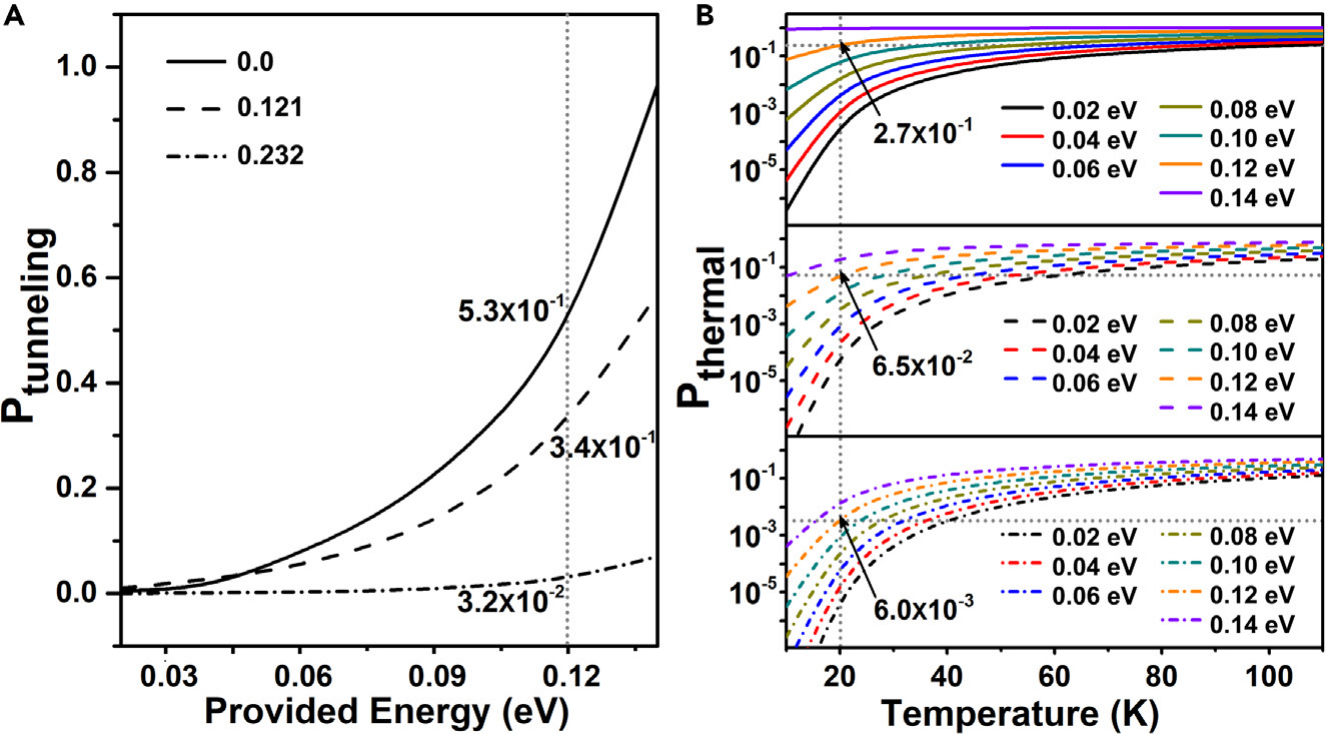
The analyses of quantum tunneling (QT) and thermal disturbance with methane rotation around Z_2_ (A) The QT probability (P_tunneling_) as a function of provided energy (E_p_) The dotted line represents the location where the E_p_ is 0.12 eV. (B) Thermal disturbance probability (P_thermal_) with temperature. The vertical dotted line represents the location where the temperature is 20 K. The horizontal dotted lines display the vertical axis value where the vertical dotted line intersects the and Pthermal curves with E_p_ of 0.12 eV.

The confined conditions may lead to strong tunneling effects from previous studies,^48^^,^^61^^,^^62^ which affect the state of the system. Not to mention the methane with light elements in this work. Thus, quantum tunneling (QT) needs to be taken seriously. It is noticed that methane rotation in this system has two distinctive features. On one hand, the energy barrier of this process is less than 0.1 eV, on the other hand, the rotational behavior can be seen as a model that the preservation of the center C atom and the rotation of the small-mass H atoms in space position. To better understand the role of QT effects, we next focus on tunneling analysis for the rotation behaviors around the Z_2_ axis due to the presence of potential well for Z_2_. The energy barriers of H atoms rotation around Z_2_ as illustrated by E_int_ are 0.014, 0.017, and 0.020 eV. For these three processes, we calculated their QT probability (P_tunneling_) under the different provided energy (E_p_), as shown in Figure 5A. With the increase of E_p_, P_tunneling_ increases gradually. The high tunneling probability is represented in the rotation process, meaning the process can easily occur through tunneling. Taking E_p_ of 0.12 eV as an example, when the $\epsilon_{V}$ is 0.0, 0.121, and 0.232, the P_tunneling_ of methane rotation is as high as 5 × 10^−1^, 3 × 10^−1^, 3 × 10^−2^, respectively. Meanwhile, the thermal disturbance probability (P_thermal_) for these processes was also studied (Figure 5B). It is found that P_thermal_ is already very high at above temperature of 100 K, while through the P_thermal_ is low at low temperature, such as at 20 K, the P_thermal_ for the $\epsilon_{V}$ of 0.0 (2.8 × 10^−1^), 0.121 (6.5 × 10^−1^), 0.232 (6.0 × 10^−3^) is smaller than P_tunneling_ at the E_p_ of 0.12 eV. These results are thus concluded that H atoms of methane are freedom in fullerene cage, and free rotation can be achieved through QT in situations where thermodynamics cannot work at low temperature. This also validates the result of the relatively free rotation of methane obtained from the experimental electron density analysis^46^ and achieves the quantum barrier-free mechanism in the system of methane under fullerene confinement.

### Conclusions

In summary, we have theoretical predicted superatomic properties of CH_4_@C_60_ under pressure by in-depth electronic analysis. Under pressure, there is the generation of new SAMOs, especially, the change of the molecular orbitals contributed by methane from regular MOs to SAMOs, when the distance between CH_4_ and C_60_ decreases within the van der Waals boundary. At this time, the methane can rotate to change the symmetry of system. The results show that the superatomic property, atom-like orbitals, are preserved during compression and dynamic rotation, which realize the extension of superatomic state under high pressure from superatomic structure of ambient condition. Moreover, the rotation of methane is freedom in the fullerene cage, even it can be achieved through QT in situations where thermodynamics play a weak role at low temperature. Our findings may provide a perspective to explain the new physical and chemical properties of superatoms in the compression process, and will stimulate the further expansion of the research on superatomic problems.

## STAR★Methods

### Key resources table

**Table:** 

REAGENT or RESOURCE	SOURCE	IDENTIFIER
**Software and algorithms**		

ADF	https://www.scm.com	N/A
Gaussian 16	http://gaussian.com/	N/A

### Resource availability

#### Lead contact

Further information and requests for resources should be directed to and will be fulfilled by the Lead Contact, Z.G. Wang (wangzg@jlu.edu.cn).

#### Materials availability

This study did not generate new unique reagents.

### Method details

#### DFT calculations

The studied system of CH_4_@C_60_ was fully optimized by using the third-generation dispersion-corrected B3LYP functional (B3LYP-D3)^63^ with the DZP^64^ basis set. To simulate isotropic hydrostatic compression resulting from pressure transmitting fluid of experiment, the equal proportional compression of fullerenes was explored. The volume strain ($\epsilon_{V}$) is defined as 1-r^3^/r_0_^3^, which varies from 0.0 to 0.232 upon compression. Subsequently, the restricted optimization was carried out under the premise of maintaining the same symmetry as the uncompressed system and fixing all carbon atoms on C_60_. In addition, to study the methane relatively rotation behaviors, interaction (E_int_) between C_60_ and CH_4_ and its four components of Pauli repulsion, electrostatic, orbital and dispersion interactions as function of rotational angle were obtained by energy decomposition analysis (EDA). The above calculations were performed using the ADF program.^65^

#### The calculations of physical properties

The physical properties being of concern on fullerene under high-pressure, the effective strain energy constant (E″), bulk modulus (B) and bond force constant (k_r_) for the CH_4_@C_60_ were calculated. Firstly, the relation of the E_S_ with respect to the $\epsilon_{V}$ could be almost perfectly regressed into a three-coefficient cubic polynomial: E_S_($\epsilon_{V}$) = a $\epsilon_{V}$ +b $\epsilon_{V}$^2^ + c $\epsilon_{V}$^3^. where the values of coefficients a, b and c were determined by regression. Then the strain energy follows the classical elasticity theory stating that the strain energy is proportional to the square of the strain. At the small strains, the strain energy in our results can be approximated by a quadratic function. E_S_ ($\epsilon_{V}$) is given by:

$$E_{S}\left(\epsilon_{V}\right)=\frac{1}{2}E^{\prime \prime }\varepsilon_{V}^{2}$$

Where the E is defined by the effective strain energy constant (or the elastic stiffness constant) per atom (E = kV_0_^2^/N, k is the spring constant), that is the second derivative of the strain energy with respect to the volume strain.

Then, the bulk modulus (B) calculated by the he relationship between the total molecular energy (E) and the volume change (ΔV) around the equilibrium volume (V_0_):^66^^,^^67^^,^^68^

$$B=V{\left(\frac{\partial^{2}E}{\partial V^{2}}\right)}_{V_{0}}$$

The relation between the fraction of energy corresponding to each individual C-C bond in the fullerene, i.e. the binding energy (E_b_) and variation of bond length (L_b_, b = 1,2) was obtained and fitted to a polynomial equation (E_1_ = −25.680L_1_^3^ + 135.500L_1_^2^-228.000L_1_+117.300, E_2_ = −22.590L_2_^3^ + 124.300L_2_^2^-218.400L_2_+117.300) for the CH_4_@C_60_. For the C_60_, E_b_ was fitted as a functional of L_b_ as a polynomial equation (E_1_ = −29.22L_1_^3^ + 151.400L_1_^2^-254.600L_1_+133.500; E_2_ = −33.240L_2_^3^ + 165.000L_2_^2^-265.800 L_2_+133.500). And the corresponding bond force constant k_rb_ was calculated by differentiation, evaluating at the corresponding binding energy minimum at $L_{b}^{0}$:^67^^,^^68^

$$k_{rb}={\left(\frac{\partial^{2}E_{b}}{\partial L_{b}^{2}}\right)}_{L_{b}^{0}}\text{.}$$

The k_r_ (=2/3 k_r1_+1/3 k_r2_) for CH_4_@C_60_ and C_60_ were calculated.

#### The calculations of tunneling probability

The quantum tunneling probability (P_tunneling_) and thermal disturbance probability (P_thermal_) of methane rotation are analyzed. The P _tunneling_ is obtained by Wenzel-Kramers-Brillouin (WKB) approximation, satisfying:

$$P_{tunneling}=\mathrm{Exp}[\frac{-2}{\hbar }\int_{x_{1}}^{x_{2}}\sqrt{2m\left(V\left(x\right)-E_{p}\right)}dx$$

which is only relevant to E_p_ for a certain potential energy surface V(x). The x_1_ and x_2_ in formula are the abscissas of the two intersection points for E = E_p_ and E = V(x), respectively. The P_thermal_ suits the Boltzmann distribution:^69^

$$P_{thermal}=\mathrm{Exp}\left(-\frac{\Delta E}{KT}\right)$$

varying with E_p_ and temperature T, where ΔE is the difference from E_p_ to the barrier (E_b_).

### Quantification and statistical analysis

Analyses and plots were performed with Microsoft Excel, PowerPoint and MATLAB.

## Data Availability

Data - Data reported in this paper will be shared by the lead contact upon request. Code - No new code was generated during the course of this study. Other - Any additional information required to reanalyze the data reported in this paper is available from the lead contact upon request.
