# Supplementary material for: Superatomic states under high pressure
Source: iScience. 2023 Mar 1;26(4):106281. doi: 10.1016/j.isci.2023.106281 (PMC10025982; doi:10.1016/j.isci.2023.106281)
Supplement: Document S1. Figures S1–S7 and Tables S1–S6 [file mmc1.pdf]

iScience, Volume *26*

Supplemental information

Superatomic states under high pressure

Rui Wang, Xinrui Yang, Wanrong Huang, Zhonghua Liu, Yu Zhu, Hanyu Liu, and Zhigang Wang

Supporting Information

Superatomic states under high pressure

Rui Wang, Xinrui Yang, Wanrong Huang, Zhonghua Liu, Yu Zhu, Hanyu Liu and Zhigang Wang

Table S1. Atomic coordinates for $CH_4@C_{60}$. Related to Figure 1(a).

Element	Number	x (Å)	y (Å)	z (Å)
C	1	0.0178	1.4264	3.2588
C	2	1.2264	0.7286	3.2588
C	3	0.0178	-1.4264	3.2588
C	4	-1.2442	-0.6978	3.2588
C	5	-1.2442	0.6978	3.2588
C	6	-0.1420	-2.6016	2.4132
C	7	-1.5007	-2.5993	1.8926
C	8	-1.7495	-3.0302	0.5899
C	9	-2.6910	-2.3045	-0.2501
C	10	-3.3466	-1.1780	0.2475
C	11	-3.0829	-0.7276	1.6070
C	12	-2.1821	-1.4238	2.4132
C	13	2.3241	-1.1779	2.4132
C	14	2.1716	-2.3061	1.6070
C	15	0.9113	-3.0337	1.6070
C	16	1.2264	-0.7286	3.2588
C	17	-3.0829	0.7276	1.6070
C	18	-2.1821	1.4238	2.4132
C	19	0.6531	-3.4872	0.2475
C	20	-0.6503	-3.4827	-0.2501
C	21	-0.9107	-3.0339	-1.6104
C	22	-2.1721	-2.3056	-1.6104
C	23	-3.5115	0.0000	-0.5942
C	24	-3.3466	1.1780	0.2475
C	25	-0.1420	2.6016	2.4132
C	26	-1.5007	2.5993	1.8926
C	27	2.3241	1.1779	2.4132
C	28	3.0015	0.0000	1.8926
C	29	2.6935	-2.3092	0.2475
C	30	1.7558	-3.0411	-0.5942
C	31	3.3413	1.1782	-0.2501
C	32	2.6935	2.3092	0.2475
C	33	1.5064	2.6092	-1.8986
C	34	2.1845	1.4277	-2.4164
C	35	3.0828	0.7283	-1.6104
C	36	0.1442	2.6057	-2.4164
C	37	-0.0174	1.4252	-3.2524
C	38	-1.2255	0.7277	-3.2524
C	39	-1.2255	-0.7277	-3.2524
C	40	-0.0174	-1.4252	-3.2524
C	41	1.2430	-0.6975	-3.2524
C	42	1.2430	0.6975	-3.2524
C	43	0.6531	3.4872	0.2475
C	44	-0.6503	3.4827	-0.2501
C	45	-0.9107	3.0339	-1.6104
C	46	1.7558	3.0411	-0.5942
C	47	2.1845	-1.4277	-2.4164
C	48	3.0828	-0.7283	-1.6104
C	49	-2.1721	2.3056	-1.6104
C	50	-2.3287	1.1780	-2.4164
C	51	-3.0128	0.0000	-1.8986
C	52	-2.3287	-1.1780	-2.4164
C	53	0.1442	-2.6057	-2.4164
C	54	1.5064	-2.6092	-1.8986
C	55	3.4990	0.0000	0.5899
C	56	3.3413	-1.1782	-0.2501
C	57	2.1716	2.3061	1.6070
C	58	0.9113	3.0337	1.6070

C	59	-1.7495	3.0302	0.5899
C	60	-2.6910	2.3045	-0.2501
C	61	0.0000	0.0000	0.0000
H	62	0.5111	-0.8853	-0.3615
H	63	0.0000	0.0000	1.0843
H	64	0.5111	0.8853	-0.3615
H	65	-1.0222	0.0000	-0.3615

Table S2. The charge on each atom of $CH_4@C_{60}$ during compression from Voronoi deformation density (VDD) charge distribution analysis. Related to Figure 1(d).

Number	0.0	0.042	0.082	0.121	0.160	0.197	0.232
1	-0.0170	-0.0190	-0.0200	-0.0210	-0.0220	-0.0250	-0.0270
2	-0.0170	-0.0190	-0.0200	-0.0210	-0.0220	-0.0250	-0.0270
3	-0.0170	-0.0190	-0.0200	-0.0210	-0.0220	-0.0250	-0.0270
4	-0.0170	-0.0190	-0.0200	-0.0210	-0.0220	-0.0250	-0.0270
5	-0.0170	-0.0190	-0.0200	-0.0210	-0.0220	-0.0250	-0.0270
6	-0.0070	-0.0080	-0.0080	-0.0090	-0.0100	-0.0080	-0.0080
7	-0.0060	-0.0060	-0.0070	-0.0090	-0.0090	-0.0120	-0.0130
8	-0.0040	-0.0030	-0.0030	-0.0040	-0.0040	-0.0050	-0.0060
9	-0.0090	-0.0110	-0.0120	-0.0130	-0.0140	-0.0140	-0.0150
10	-0.0130	-0.0150	-0.0160	-0.0170	-0.0190	-0.0170	-0.0180
11	-0.0070	-0.0060	-0.0070	-0.0080	-0.0080	-0.0100	-0.0120
12	-0.0070	-0.0080	-0.0080	-0.0090	-0.0100	-0.0080	-0.0080
13	-0.0070	-0.0080	-0.0080	-0.0090	-0.0100	-0.0080	-0.0080
14	-0.0070	-0.0060	-0.0070	-0.0080	-0.0080	-0.0100	-0.0120
15	-0.0070	-0.0060	-0.0070	-0.0080	-0.0080	-0.0100	-0.0120
16	-0.0170	-0.0190	-0.0200	-0.0210	-0.0220	-0.0250	-0.0270
17	-0.0070	-0.0060	-0.0070	-0.0080	-0.0080	-0.0100	-0.0120
18	-0.0070	-0.0080	-0.0080	-0.0090	-0.0100	-0.0080	-0.0080
19	-0.0130	-0.0150	-0.0160	-0.0170	-0.0190	-0.0170	-0.0180
20	-0.0090	-0.0110	-0.0120	-0.0130	-0.0140	-0.0140	-0.0150
21	-0.0100	-0.0100	-0.0110	-0.0110	-0.0120	-0.0150	-0.0170
22	-0.0100	-0.0100	-0.0110	-0.0110	-0.0120	-0.0150	-0.0170
23	-0.0170	-0.0200	-0.0210	-0.0220	-0.0240	-0.0230	-0.0240
24	-0.0130	-0.0150	-0.0160	-0.0170	-0.0190	-0.0170	-0.0180
25	-0.0070	-0.0080	-0.0080	-0.0090	-0.0100	-0.0080	-0.0080
26	-0.0060	-0.0060	-0.0070	-0.0090	-0.0090	-0.0120	-0.0130
27	-0.0070	-0.0080	-0.0080	-0.0090	-0.0100	-0.0080	-0.0080
28	-0.0060	-0.0060	-0.0070	-0.0090	-0.0090	-0.0120	-0.0130
29	-0.0130	-0.0150	-0.0160	-0.0170	-0.0190	-0.0170	-0.0180
30	-0.0170	-0.0200	-0.0210	-0.0220	-0.0240	-0.0230	-0.0240
31	-0.0090	-0.0110	-0.0120	-0.0130	-0.0140	-0.0140	-0.0150
32	-0.0130	-0.0150	-0.0160	-0.0170	-0.0190	-0.0170	-0.0180
33	-0.0260	-0.0270	-0.0290	-0.0310	-0.0330	-0.0380	-0.0410
34	-0.0130	-0.0140	-0.0150	-0.0170	-0.0190	-0.0190	-0.0200
35	-0.0100	-0.0100	-0.0110	-0.0110	-0.0120	-0.0150	-0.0170
36	-0.0130	-0.0140	-0.0150	-0.0170	-0.0190	-0.0190	-0.0200
37	-0.0060	-0.0060	-0.0070	-0.0070	-0.0070	-0.0090	-0.0100
38	-0.0060	-0.0060	-0.0070	-0.0070	-0.0070	-0.0090	-0.0100
39	-0.0060	-0.0060	-0.0070	-0.0070	-0.0070	-0.0090	-0.0100
40	-0.0060	-0.0060	-0.0070	-0.0070	-0.0070	-0.0090	-0.0100
41	-0.0060	-0.0060	-0.0070	-0.0070	-0.0070	-0.0090	-0.0100
42	-0.0060	-0.0060	-0.0070	-0.0070	-0.0070	-0.0090	-0.0100
43	-0.0130	-0.0150	-0.0160	-0.0170	-0.0190	-0.0170	-0.0180
44	-0.0090	-0.0110	-0.0120	-0.0130	-0.0140	-0.0140	-0.0150
45	-0.0100	-0.0100	-0.0110	-0.0110	-0.0120	-0.0150	-0.0170
46	-0.0170	-0.0200	-0.0210	-0.0220	-0.0240	-0.0230	-0.0240
47	-0.0130	-0.0140	-0.0150	-0.0170	-0.0190	-0.0190	-0.0200
48	-0.0100	-0.0100	-0.0110	-0.0110	-0.0120	-0.0150	-0.0170
49	-0.0100	-0.0100	-0.0110	-0.0110	-0.0120	-0.0150	-0.0170
50	-0.0130	-0.0140	-0.0150	-0.0170	-0.0190	-0.0190	-0.0200
51	-0.0260	-0.0270	-0.0290	-0.0310	-0.0330	-0.0380	-0.0410
52	-0.0130	-0.0140	-0.0150	-0.0170	-0.0190	-0.0190	-0.0200
53	-0.0130	-0.0140	-0.0150	-0.0170	-0.0190	-0.0190	-0.0200
54	-0.0260	-0.0270	-0.0290	-0.0310	-0.0330	-0.0380	-0.0410
55	-0.0040	-0.0030	-0.0030	-0.0040	-0.0040	-0.0050	-0.0060
56	-0.0090	-0.0110	-0.0120	-0.0130	-0.0140	-0.0140	-0.0150
57	-0.0070	-0.0060	-0.0070	-0.0080	-0.0080	-0.0100	-0.0120

58	-0.0070	-0.0060	-0.0070	-0.0080	-0.0080	-0.0100	-0.0120
59	-0.0040	-0.0030	-0.0030	-0.0040	-0.0040	-0.0050	-0.0060
60	-0.0090	-0.0110	-0.0120	-0.0130	-0.0140	-0.0140	-0.0150
61	-0.0970	-0.1000	-0.1020	-0.1040	-0.1080	-0.1100	-0.1130
62	0.1870	0.2000	0.2140	0.2300	0.2450	0.2630	0.2820
63	0.1860	0.1990	0.2140	0.2290	0.2440	0.2620	0.2810
64	0.1870	0.2000	0.2140	0.2300	0.2450	0.2630	0.2820
65	0.1870	0.2000	0.2140	0.2300	0.2450	0.2630	0.2820

Table S3. The charge on each atom of $CH_4@C_{60}$ during compression from Hirshfeld charge distribution analysis. Related to Figure 1(d).

Number	0.0	0.042	0.082	0.121	0.160	0.197	0.232
1	-0.0004	-0.0006	-0.0008	-0.0011	-0.0014	-0.0017	-0.0021
2	-0.0004	-0.0006	-0.0008	-0.0011	-0.0014	-0.0017	-0.0021
3	-0.0004	-0.0006	-0.0008	-0.0011	-0.0014	-0.0017	-0.0021
4	-0.0004	-0.0006	-0.0008	-0.0011	-0.0014	-0.0017	-0.0021
5	-0.0004	-0.0006	-0.0008	-0.0011	-0.0014	-0.0017	-0.0021
6	0.0006	0.0004	0.0003	0.0001	0.0000	-0.0002	-0.0003
7	0.0013	0.0012	0.0010	0.0009	0.0009	0.0007	0.0004
8	0.0010	0.0009	0.0008	0.0007	0.0006	0.0005	0.0004
9	0.0009	0.0009	0.0007	0.0006	0.0005	0.0003	0.0001
10	-0.0004	-0.0007	-0.0009	-0.0012	-0.0013	-0.0016	-0.0018
11	0.0006	0.0005	0.0003	0.0001	0.0000	-0.0002	-0.0006
12	0.0006	0.0004	0.0003	0.0001	0.0000	-0.0002	-0.0003
13	0.0006	0.0004	0.0003	0.0001	0.0000	-0.0002	-0.0003
14	0.0006	0.0005	0.0003	0.0001	0.0000	-0.0002	-0.0006
15	0.0006	0.0005	0.0003	0.0001	0.0000	-0.0002	-0.0006
16	-0.0004	-0.0006	-0.0008	-0.0011	-0.0014	-0.0017	-0.0021
17	0.0006	0.0005	0.0003	0.0001	0.0000	-0.0002	-0.0006
18	0.0006	0.0004	0.0003	0.0001	0.0000	-0.0002	-0.0003
19	-0.0004	-0.0007	-0.0009	-0.0012	-0.0013	-0.0016	-0.0018
20	0.0009	0.0009	0.0007	0.0006	0.0005	0.0003	0.0001
21	0.0006	0.0005	0.0003	0.0001	0.0000	-0.0002	-0.0006
22	0.0006	0.0005	0.0003	0.0001	0.0000	-0.0002	-0.0006
23	-0.0007	-0.0009	-0.0012	-0.0015	-0.0018	-0.0022	-0.0023
24	-0.0004	-0.0007	-0.0009	-0.0012	-0.0013	-0.0016	-0.0018
25	0.0006	0.0004	0.0003	0.0001	0.0000	-0.0002	-0.0003
26	0.0013	0.0012	0.0010	0.0009	0.0009	0.0007	0.0004
27	0.0006	0.0004	0.0003	0.0001	0.0000	-0.0002	-0.0003
28	0.0013	0.0012	0.0010	0.0009	0.0009	0.0007	0.0004
29	-0.0004	-0.0007	-0.0009	-0.0012	-0.0013	-0.0016	-0.0018
30	-0.0007	-0.0009	-0.0012	-0.0015	-0.0018	-0.0022	-0.0023
31	0.0009	0.0009	0.0007	0.0006	0.0005	0.0003	0.0001
32	-0.0004	-0.0007	-0.0009	-0.0012	-0.0013	-0.0016	-0.0018
33	-0.0009	-0.001	-0.0013	-0.0015	-0.0018	-0.0022	-0.0025
34	0.0000	-0.0003	-0.0005	-0.0007	-0.0009	-0.0012	-0.0013
35	0.0006	0.0005	0.0003	0.0001	0.0000	-0.0002	-0.0006
36	0.0000	-0.0003	-0.0005	-0.0007	-0.0009	-0.0012	-0.0013
37	0.0008	0.0006	0.0005	0.0003	0.0003	0.0001	-0.0002
38	0.0008	0.0006	0.0005	0.0003	0.0003	0.0001	-0.0002
39	0.0008	0.0006	0.0005	0.0003	0.0003	0.0001	-0.0002
40	0.0008	0.0006	0.0005	0.0003	0.0003	0.0001	-0.0002
41	0.0008	0.0006	0.0005	0.0003	0.0003	0.0001	-0.0002
42	0.0008	0.0006	0.0005	0.0003	0.0003	0.0001	-0.0002
43	-0.0004	-0.0007	-0.0009	-0.0012	-0.0013	-0.0016	-0.0018
44	0.0009	0.0009	0.0007	0.0006	0.0005	0.0003	0.0001
45	0.0006	0.0005	0.0003	0.0001	0.0000	-0.0002	-0.0006
46	-0.0007	-0.0009	-0.0012	-0.0015	-0.0018	-0.0022	-0.0023
47	0.0000	-0.0003	-0.0005	-0.0007	-0.0009	-0.0012	-0.0013
48	0.0006	0.0005	0.0003	0.0001	0.0000	-0.0002	-0.0006
49	0.0006	0.0005	0.0003	0.0001	0.0000	-0.0002	-0.0006
50	0.0000	-0.0003	-0.0005	-0.0007	-0.0009	-0.0012	-0.0013
51	-0.0009	-0.0010	-0.0013	-0.0015	-0.0018	-0.0022	-0.0025
52	0.0000	-0.0003	-0.0005	-0.0007	-0.0009	-0.0012	-0.0013
53	0.0000	-0.0003	-0.0005	-0.0007	-0.0009	-0.0012	-0.0013
54	-0.0009	-0.001	-0.0013	-0.0015	-0.0018	-0.0022	-0.0025
55	0.0010	0.0009	0.0008	0.0007	0.0006	0.0005	0.0004
56	0.0009	0.0009	0.0007	0.0006	0.0005	0.0003	0.0001
57	0.0006	0.0005	0.0003	0.0001	0.0000	-0.0002	-0.0006

58	0.0006	0.0005	0.0003	0.0001	0.0000	-0.0002	-0.0006
59	0.0010	0.0009	0.0008	0.0007	0.0006	0.0005	0.0004
60	0.0009	0.0009	0.0007	0.0006	0.0005	0.0003	0.0001
61	-0.1357	-0.1334	-0.1298	-0.126	-0.1258	-0.1209	-0.1159
62	0.0296	0.0311	0.033	0.0352	0.0369	0.0395	0.0422
63	0.0295	0.0311	0.0331	0.0352	0.0369	0.0394	0.0421
64	0.0296	0.0311	0.033	0.0352	0.0369	0.0395	0.0422
65	0.0296	0.0311	0.033	0.0352	0.0369	0.0395	0.0422

Figure S1. Five-fold degenerate vibrational modes of Hg(8) by calcuated the vibration-related Raman spectrums at the M06-D3 [1,2] in conjunction with 6-31+G* for C_{60} and 6-31+G** basis sets [3,4] for CH_4 level using Gaussian 16 program [5]. Related to Figure 1(f).

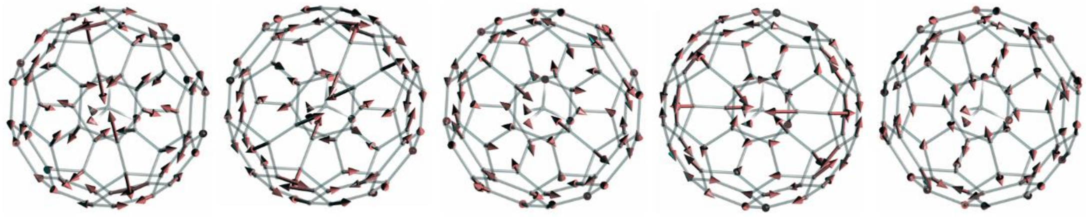

Figure S2. Raman spectrum of isolated C_{60} during compression using a numerical frequency correction factor of value 0.989 [6]. Related to Figure 1 (f).

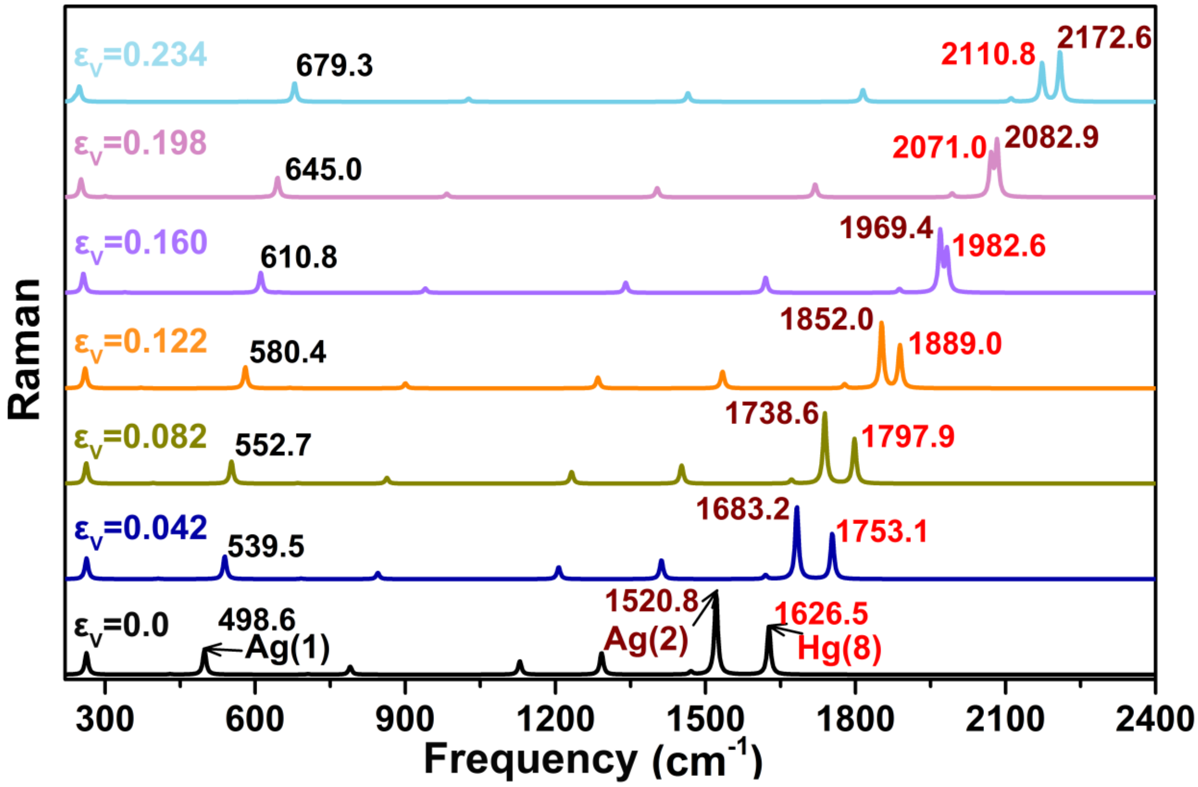

Figure S3. The MOs energy levels about the unoccupied orbitals during the compression for $CH_4@C_{60}$. The purple and red lines respectively represent the S and P SAMOs, which are concern on in provious report [7]. Related to Figure 2.

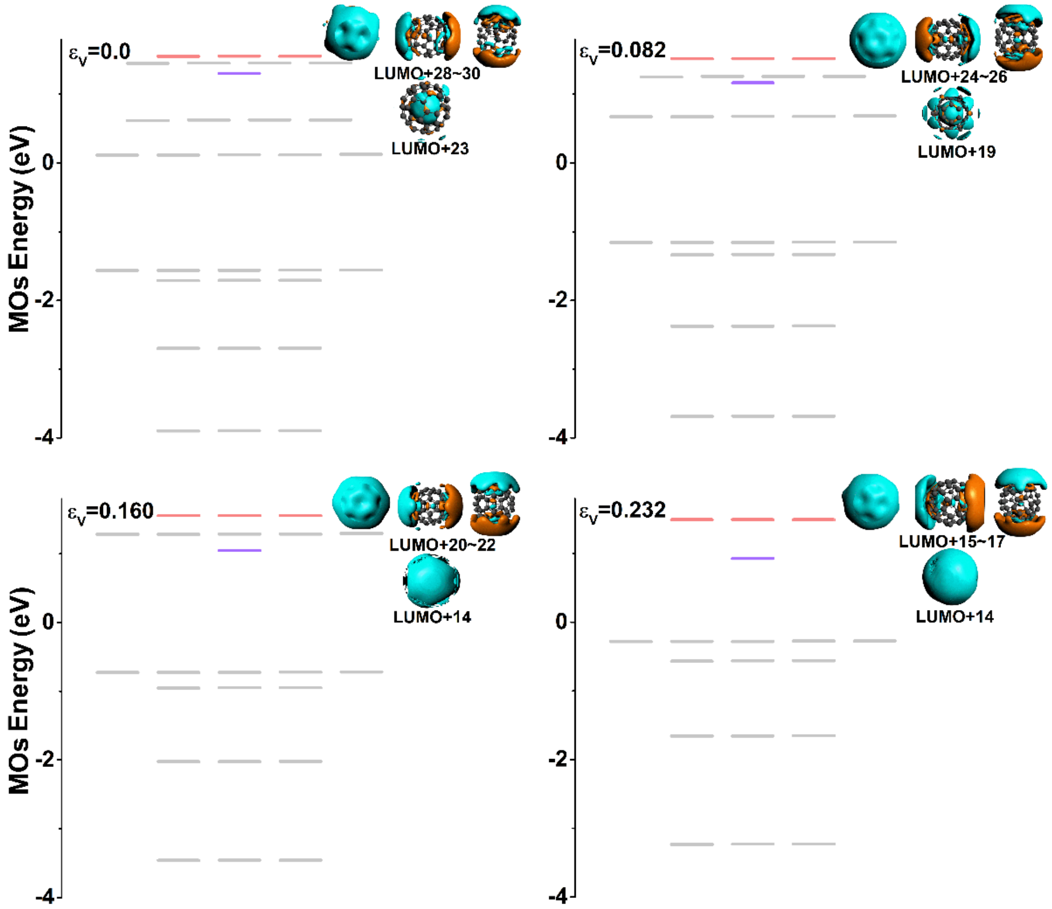

Table S4. The components ratios of S and P SAMOs for $CH_4@C_{60}$. Related to Figure 2.

	A1 (S SAMO)		A1 (P SAMO)			E1 (P SAMOs)		
ε_V [a]	f1 [b]	f2 [b]	f1-1 [c]	f1-2 [c]	f2	f1-1	f1-2	f2
0.0	6.91% [3]	94.52%	73.51%	5.97%	20.24%	73.49%	5.89%	20.19%
0.082	12.17%	90.05%	68.48%	7.82%	23.44%	68.50%	7.80%	23.43%
0.160	17.29%	86.88%	89.80%		5.28%	89.81%		5.29%
0.232	21.18%	84.12%	89.00%		5.39%	89.04%		5.43%

[a] Since less than 1% of the orbital components are not accounted, and the sum of counted components is not exactly 100%.

[b] The f1 and f2 display the C_{60} cage and CH_4 molecule.

[c] The f1-1 and f1-2 show that orbital origin is different.

Figure S4**.** The energy levels diagrams during compression. Related to Figure 2.

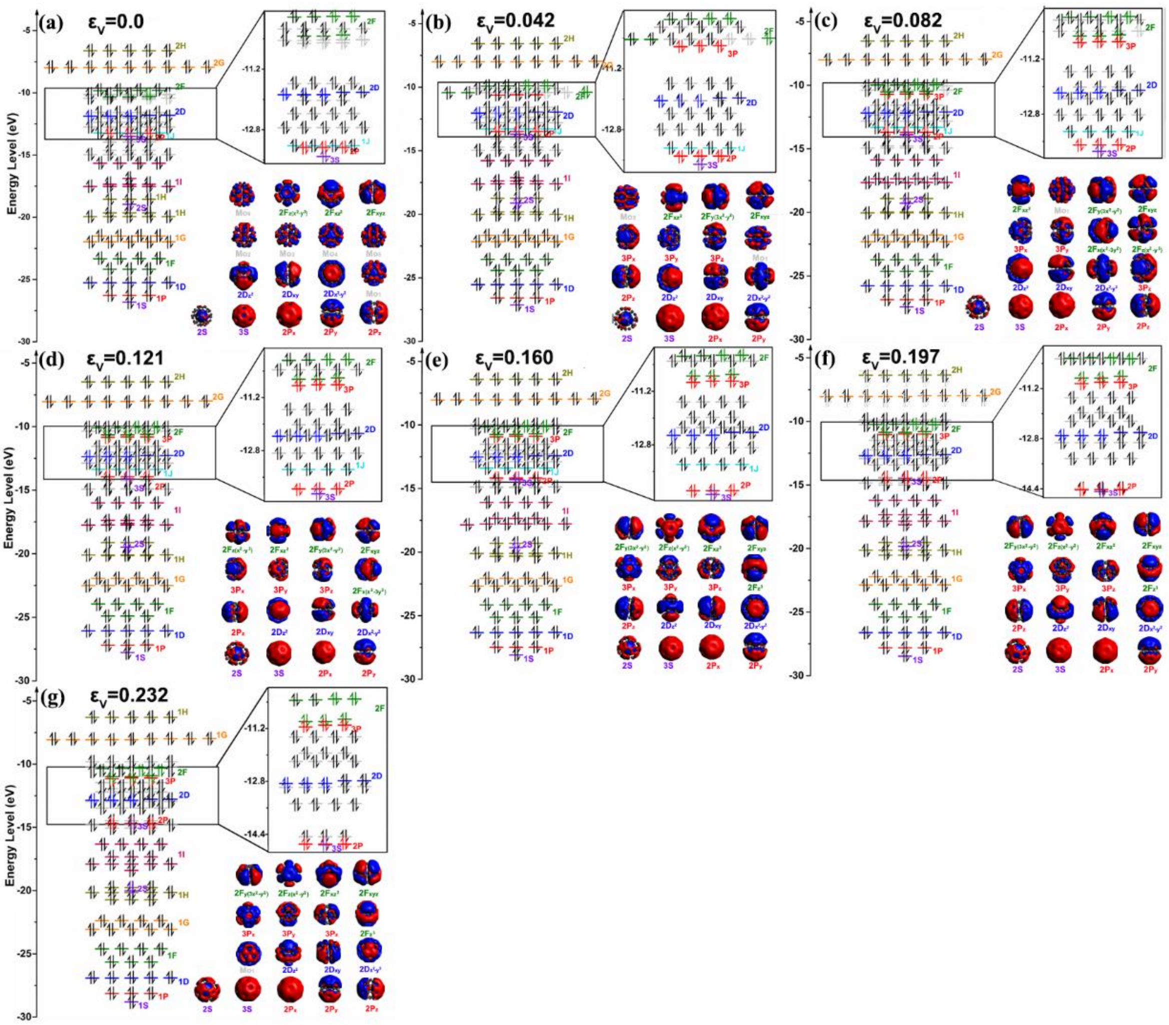

Table S5. The components ratios of MOs highlighted in the Figure 2. Related to Figure 2.

0.0			0.121			0.232		
MOs	f1[a]	f2[a]	MOs	f1	f2	MOs	f1	f2
$2F_{xyz}$	98.60%[b]	1.28%	$2F_{xyz}$	98.29%	1.58%	$2F_{xyz}$	97.39%	2.05%
$2F_{xz^2}$	98.60%	1.28%	$2F_{xz^2}$	98.29%	1.58%	$2F_{xz^2}$	97.39%	2.05%
$2F_{z(x^2-y^2)}$	85.95%	12.87%	$2F_{z^3}$	83.96%	15.19%	$2F_{z^3}$	72.69%	25.63%
MO1	78.10%	19.58%	$2F_{z(x^2-y^2)}$	98.53%	1.09%	$2F_{z(x^2-y^2)}$	95.32%	3.49%
MO2	78.10%	19.58%	$2F_{y(3x^2-y^2)}$	98.53%	1.09%	$2F_{y(3x^2-y^2)}$	95.32%	3.49%
MO3	89.58%	9.55%	$3P_y$	31.93%	79.29%	$3P_y$	23.50%	73.52%
MO4	35.28%	63.36%	$3P_x$	31.93%	79.29%	$3P_x$	23.50%	73.52%
MO5	35.28%	63.36%	$3P_z$	19.28%	66.67%	$3P_z$	45.72%	52.96%
MO6	21.58%	62.54%	$2D_{z^2}$	96.08%	3.01%	$2D_{z^2}$	97.19%	2.06%
$2D_{z^2}$	96.52%	2.83%	$2D_{xy}$	95.93%	3.10%	$2D_{xy}$	96.42%	2.08%
$2D_{xy}$	96.49%	2.85%	$2D_{x^2-y^2}$	95.93%	3.10%	$2D_{x^2-y^2}$	96.42%	2.08%
$2D_{x^2-y^2}$	96.49%	2.85%	$2P_z$	85.56%	13.65%	MO1	98.57%	1.01%
$2P_z$	87.50%	11.51%	$2P_y$	85.51%	13.69%	$2P_z$	83.70%	15.16%
$2P_y$	87.24%	11.46%	$2P_x$	85.51%	13.69%	$2P_y$	83.77%	15.12%
$2P_x$	87.24%	11.46%	3S	96.07%	2.62%	$2P_x$	83.77%	15.12%
3S	97.55%	1.70%				3S	94.61%	4.12%

[a]The f1 and f2 display the C_{60} cage and CH_4 molecule.

[b]Since less than 1% of the orbital components are not accounted, the sum of f1 and f2 is not exactly 100%.

Figure S5. The diagrams of density of states (DOS) during compression. Related to Figure 2.

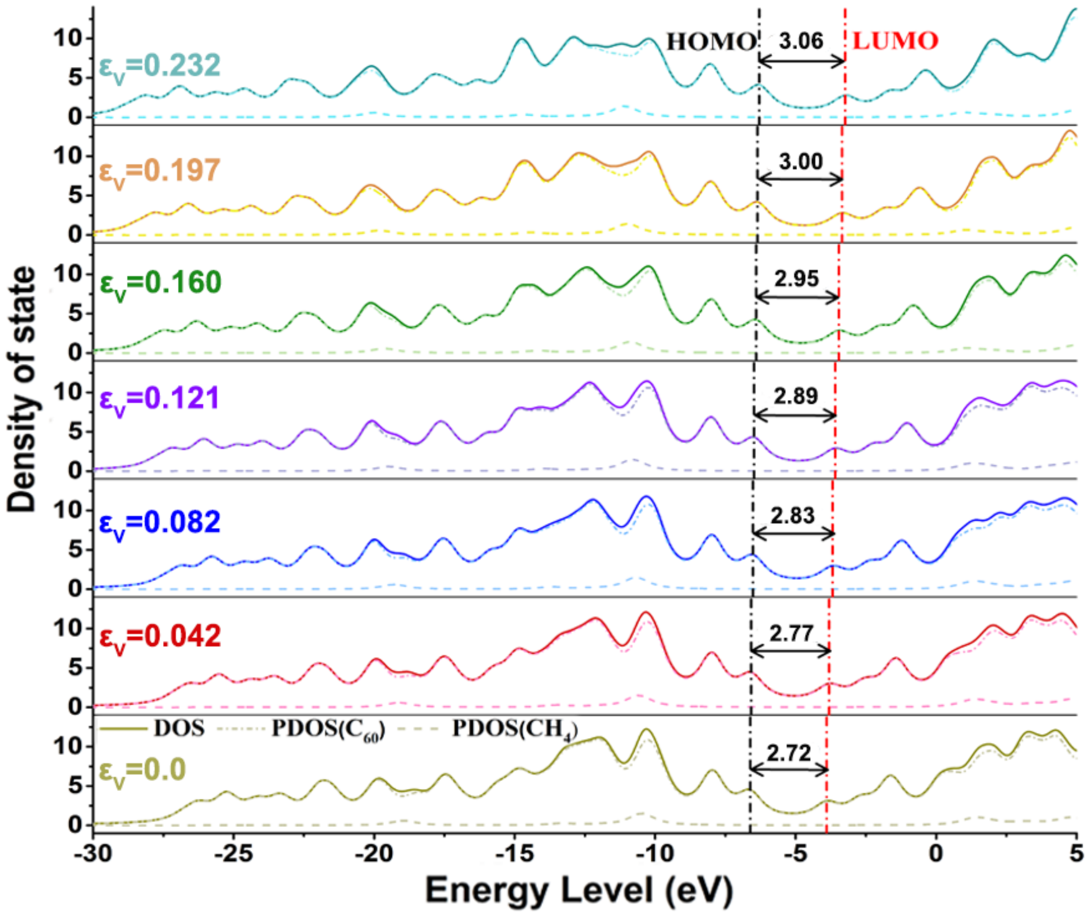

Figure S6. The ultraviolet-visible (UV-Vis) absorption spectrum during compression at B3LYP-D3/6-31G** level by Gaussian 16 program [3-5,8]. Related to Figure 2.

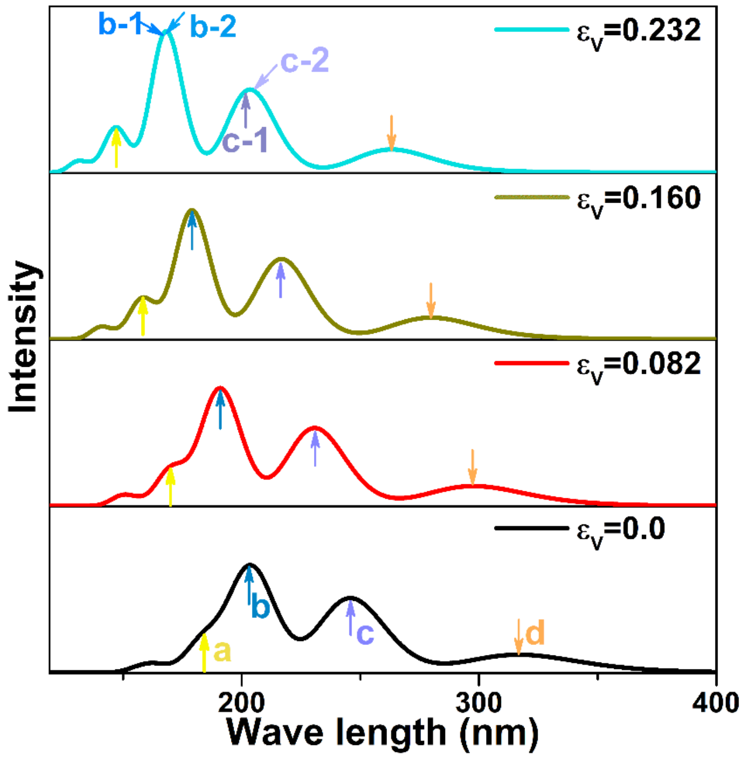

Table S6. Excitation wavelength (λ), orbital transitions details of the UV-Vis spectra during comprssion. Related to Figure 2.

ε_V[a]	Peak	E[b] (eV)	λ(nm)	f	Orbital translation	MOs type[c]	C (%)
0.0	a	6.72	184.55	0.286	H-1[d] → L+19[d]	2H→R	20.9
					H-3 → L+21	2H→R	16.0
					H-4 → L+22	2H→R	15.6
		6.72	184.55	0.286	H-2 → L+19	2H→R	21.0
					H-4 → L+21	2H→R	16.0
					H-3 → L+22	2H→R	15.8
		6.72	184.50	0.293	H → L+20	2H→R	31.8
					H-4 → L+22	2H→R	11.8
					H-3 → L+21	2H→R	11.1
					H-2 → L+21	2H→R	11.0
					H-1 → L+22	2H→R	10.7
	b	6.07	204.25	0.915	H-6 → L+6	2G→R	38.1
					H-7 → L+7	2G→R	11.3
					H-8 → L+8	2G→R	10.7
		6.07	204.20	0.917	H-5 → L+8	2G→R	22.1
					H-7 → L+8	2G→R	11.8
					H-8 → L+7	2G→R	11.8
					H-7 → L+6	2G→R	10.6
		6.07	204.20	0.917	H-5 → L+7	2G→R	21.9
					H-8 → L+8	2G→R	11.9
					H-7 → L+7	2G→R	11.6
					H-8 → L+6	2G→R	10.9
	c	5.04	245.98	0.664	H-1 → L+10	2H→R	25.4
					H-2 → L+9	2H→R	25.4
		5.04	245.98	0.664	H → L+13	2H→R	18.7
					H-3 → L+11	2H→R	18.7
		5.04	245.98	0.664	H → L+12	2H→R	19.9
					H-4 → L+11	2H→R	19.7
	d	3.92	316.17	0.158	H-10 → L+2	2G→2H	16.2
					H-9 → L+1	2G→2H	15.9
					H-12 → L	2G→2H	15.6
		3.92	316.17	0.158	H-9 → L+2	2G→2H	16.1
					H-10 → L+1	2G→2H	16.1
					H-11 → L	2G→2H	16.0
		3.92	316.12	0.157	H-13 → L	2G→2H	24.7
					H-12 → L+2	2G→2H	15.9
					H-11 → L+1	2G→2H	15.8
0.082	a	7.26	170.71	0.325	H-1 → L+19	2H→R	26.7
					H-3 → L+21	2H→R	18.1
					H-4 → L+22	2H→R	17.7
					H-3 → L+20	2H→R	12.1
		7.26	170.71	0.325	H-2 → L+19	2H→R	26.9
					H-4 → L+21	2H→R	18.2
					H-3 → L+22	2H→R	18.0
					H-4 → L+20	2H→R	11.7
		7.26	170.66	0.331	H → L+20	2H→R	39.1
					H-4 → L+22	2H→R	14.0
					H-3 → L+21	2H→R	13.3
					H-2 → L+21	2H→R	13.0
					H-1 → L+22	2H→R	12.7
	b	6.48	191.19	1.030	H-6 → L+6	2G→R	39.2
					H-7 → L+8	2G→R	11.4
					H-8 → L+7	2G→R	10.7
		6.49	191.14	1.031	H-5 → L+7	2G→R	22.6
					H-8 → L+8	2G→R	12.4

					H-7 → L+7	2G→R	12.4
					H-7 → L+6	2G→R	10.6
		6.49	191.14	1.031	H-5 → L+8	2G→R	22.4
					H-8 → L+7	2G→R	12.6
					H-7 → L+8	2G→R	12.2
					H-8 → L+6	2G→R	10.9
	c	5.36	231.00	0.692	H-1 → L+10	2H→R	25.5
					H-2 → L+9	2H→R	25.5
		5.37	230.87	0.692	H → L+13	2H→R	18.9
					H-3 → L+11	2H→R	18.6
		5.37	230.87	0.692	H-4 → L+11	2H→R	20.0
					H → L+12	2H→R	19.8
	d	4.17	297.18	0.175	H-10 → L+2	2G→2H	16.4
					H-9 → L+1	2G→2H	16.0
					H-12 → L	2G→2H	15.8
		4.17	297.18	0.175	H-9 → L+2	2G→2H	16.3
					H-10 → L+1	2G→2H	16.2
					H-11 → L	2G→2H	16.2
		4.17	297.14	0.175	H-13 → L	2G→2H	24.8
					H-12 → L+2	2G→2H	16.2
					H-11 → L+1	2G→2H	16.1
0.160	a	7.83	158.27	0.357	H-1 → L+19	2H→R	28.1
					H-3 → L+21	2H→R	17.7
					H-4 → L+22	2H→R	17.3
					H-3 → L+20	2H→R	12.8
		7.83	158.27	0.357	H-2 → L+19	2H→R	28.3
					H-4 → L+21	2H→R	17.7
					H-3 → L+22	2H→R	17.6
					H-4 → L+20	2H→R	12.8
		7.84	158.23	0.362	H → L+20	2H→R	39.8
					H-4 → L+22	2H→R	13.9
					H-3 → L+21	2H→R	13.3
					H-2 → L+21	2H→R	13.2
					H-1 → L+22	2H→R	12.9
	b	6.91	179.38	1.101	H-6 → L+6	2G→R	38.3
					H-7 → L+8	2G→R	11.0
					H-8 → L+7	2G→R	10.3
		6.91	179.33	1.101	H-5 → L+7	2G→R	22.0
					H-8 → L+8	2G→R	12.2
					H-7 → L+7	2G→R	12.2
					H-7 → L+6	2G→R	10.2
		6.91	179.33	1.101	H-5 → L+8	2G→R	21.8
					H-8 → L+7	2G→R	12.4
					H-7 → L+8	2G→R	11.9
					H-8 → L+6	2G→R	10.4
	c	5.70	217.58	0.667	H-1 → L+10	2H→R	24.3
					H-2 → L+9	2H→R	24.3
		5.70	217.45	0.669	H → L+13	2H→R	18.0
					H-3 → L+11	2H→R	17.5
		5.70	217.45	0.669	H-4 → L+11	2H→R	19.5
					H → L+12	2H→R	18.8
	d	4.44	279.48	0.191	H-10 → L+2	2G→2H	16.6
					H-9 → L+1	2G→2H	16.2
					H-12 → L	2G→2H	16.0
		4.44	279.48	0.191	H-9 → L+2	2G→2H	16.5
					H-10 → L+1	2G→2H	16.4
					H-11 → L	2G→2H	16.4
		4.44	279.44	0.190	H-13 → L	2G→2H	25.0
					H-12 → L+2	2G→2H	16.3

					H-11 → L+1	2G→2H	16.3
0.232	a	8.42	147.22	0.391	H-1 → L+19	2H→R	30.0
					H-3 → L+21	2H→R	16.6
					H-4 → L+22	2H→R	16.2
					H-3 → L+20	2H→R	13.3
		8.42	147.22	0.391	H-2 → L+19	2H→R	30.1
					H-4 → L+21	2H→R	16.7
					H-3 → L+22	2H→R	16.6
					H-4 → L+20	2H→R	12.8
		8.42	147.18	0.395	H → L+20	2H→R	40.0
					H-4 → L+22	2H→R	13.6
					H-2 → L+21	2H→R	13.1
					H-3 → L+21	2H→R	12.8
					H-1 → L+22	2H→R	12.8
	b-1	7.40	167.58	0.828	H-6 → L+6	2G→R	22.8
					H-18 → L+5	**R→2H**[e]	16.5
					H-15 → L+3	**R→2H**	10.5
					H-14 → L+4	**R→2H**	10.2
		7.40	167.56	0.851	H-5 → L+7	2G→R	11.2
					H-15 → L+5	**R→2H**	10.9
		7.40	167.56	0.851	H-5 → L+8	2G→R	11.2
					H-14 → L+5	**R→2H**	10.9
	b-2	7.31	169.61	0.427	H-18 → L+5	**R→2H**	24.8
					H-15 → L+3	**R→2H**	15.7
					H-6 → L+6	2G→R	15.6
					H-14 → L+4	**R→2H**	15.5
		7.31	169.60	0.411	H-17 → L+4	**R→2H**	15.2
					H-15 → L+5	**R→2H**	15.1
					H-16 → L+3	**R→2H**	14.8
		7.31	169.60	0.411	H-17 → L+3	**R→2H**	15.2
					H-16 → L+4	**R→2H**	14.8
					H-14 → L+5	**R→2H**	14.8
	c-1	6.16	201.11	0.427	H-22 → L+2	**R→2H**	15.6
					H-23 → L+1	**R→2H**	15.6
					H-21 → L	**R→2H**	14.2
					H-2 → L+9	2H→R	11.8
					H-1 → L+10	2H→R	11.8
		6.17	201.07	0.436	H-19 → L+1	**R→2H**	14.3
					H-22 → L	**R→2H**	13.5
					H-21 → L+2	**R→2H**	12.7
		6.17	201.07	0.436	H-20 → L+1	**R→2H**	18.9
					H-19 → L+2	**R→2H**	14.4
					H-23 → L	**R→2H**	13.7
					H-4 → L+11	2H→R	10.0
	c-2	5.99	206.94	0.347	H-21 → L	**R→2H**	16.3
					H-2 → L+9	2H→R	14.3
					H-1 → L+10	2H→R	14.2
					H-23 → L+1	**R→2H**	13.0
					H-22 → L+2	**R→2H**	13.0
		5.99	206.86	0.339	H-20 → L+1	**R→2H**	18.1
					H-23 → L	**R→2H**	14.6
					H-19 → L+2	**R→2H**	13.6
					H-4 → L+11	2H→R	11.6
					H → L+12	2H→R	10.7
		5.99	206.86	0.339	H-22 → L	**R→2H**	14.5
					H-19 → L+1	**R→2H**	13.6
					H-21 → L+2	**R→2H**	12.2
	d	4.72	262.93	0.204	H-10 → L+2	2G→2H	16.4
					H-12 → L	2G→2H	16.1

			H-9 → L+1	2G→2H	16.0
4.72	262.93	0.204	H-11 → L	2G→2H	16.6
			H-10 → L+1	2G→2H	16.5
			H-9 → L+2	2G→2H	16.2
4.72	262.88	0.203	H-13 → L	2G→2H	25.1
			H-11 → L+1	2G→2H	16.6
			H-12 → L+2	2G→2H	16.4

[a]Transitional oscillator strength (f) less than 0.1 and contributions of orbitals to the transition (C) that account for less than 10% are not included in the statistics.
[b]The E display the excited energy
[c]The “MOs type" column shows the types of excited orbitals.
[d]The H and L refer to HOMO and LUMO.
[e]The bold fonts are the source of the new orbital transitions.

Figure S7. Schematic diagram of the path projected onto the C_{60} cage during the rotation of H atoms. Related to Figure 3.

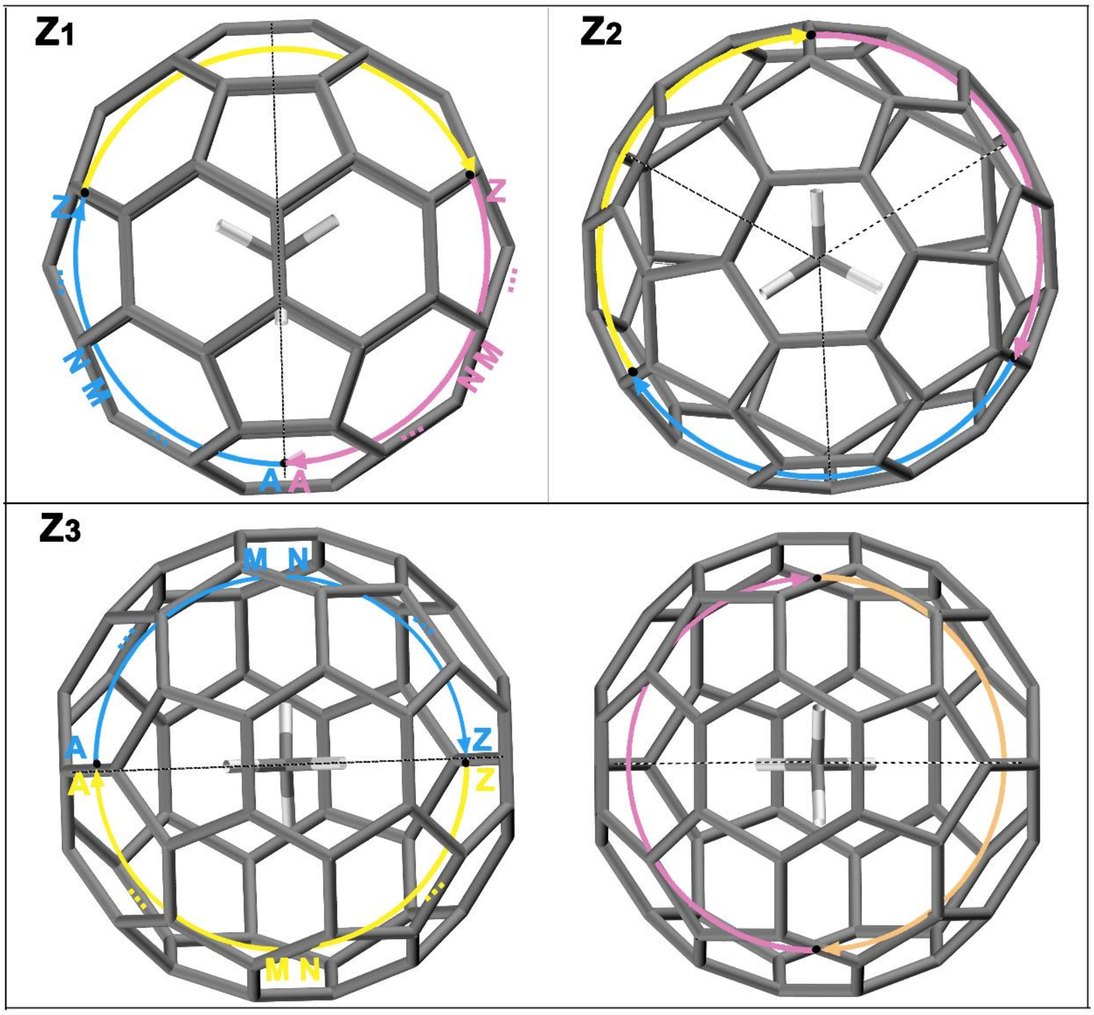

Supplemental Reference

1. Zhao, Y., and Truhlar, D.G. (2008). The M06 suite of density functionals for main group thermochemistry, thermochemical kinetics, noncovalent interactions, excited states, and transition elements: two new functionals and systematic testing of four M06 functionals and 12 other functionals. Theor. Chem. Acc. *119*, 525-525. https://doi.org/10.1007/s00214-007-0401-8.

2. Grimme, S., Antony, J., Ehrlich, S., and Krieg, H. (2010). A consistent and accurate ab initio parametrization of density functional dispersion correction (DFT-D) for the 94 elements H-Pu. J. Chem. Phys. *132*, 154104. https://doi.org/10.1063/1.3382344.

3. Francl, M., Pietro, W.J., Hehre, W.J., Binkley, J.S., Gordon, M.S., DeFrees, D.J., and Pople, J.A. (1982). Self-consistent molecular orbital methods. XXIII. A polarization-type basis set for second-row elements. J. Chem. Phys.; (United States) *77*. https://doi.org/10.1063/1.444267.

4. Hariharan, P.C., and Pople, J.A. (1973). The influence of polarization functions on molecular orbital hydrogenation energies. Theoret. Chim. Acta *28*, 213-222. https://doi.org/10.1007/bf00533485.

5. Frisch, M.J., Trucks, G.W., Schlegel, H.B., Scuseria, G.E., Robb, M.A., Cheeseman, J.R., Scalmani, G., Barone, V., Petersson, G.A., Nakatsuji, H., et al. (2016). Gaussian 16 Rev. C.01.

6. Alecu, I.M., Zheng, J., Zhao, Y., and Truhlar, D.G. (2010). Computational Thermochemistry: Scale Factor Databases and Scale Factors for Vibrational Frequencies Obtained from Electronic Model Chemistries. J. Chem. Theory. Comput. *6*, 2872-2887. https://doi.org/10.1021/ct100326h.

7. Feng, M., Zhao, J., and Petek, H. (2008). Atomlike, hollow-core-bound molecular orbitals of C_{60}. Science *320*, 359-362. https://doi.org/10.1126/science.1155866.

8. Grimme, S., Antony, J., Ehrlich, S., and Krieg, H. (2010). A consistent and accurate ab initio parametrization of density functional dispersion correction (DFT-D) for the 94 elements H-Pu. J Chem Phys *132*, 154104. https://doi.org/10.1063/1.3382344.
